# Feasibility of anticoagulation using low molecular-weight heparin during catheter-directed thrombolysis for lower extremity deep venous thrombosis

**DOI:** 10.1186/s12959-021-00260-3

**Published:** 2021-01-28

**Authors:** Yonghui Li, Junwei Wang, Rongzhou He, Junmeng Zheng, Zhibo Chen, Chen Yao, Kai Huang

**Affiliations:** 1 Department of Cardiovascular Surgery, the Sun Yat-sen Memorial Hospital of Sun Yat-sen University, 107 Yanjiang West Road, Guangzhou, 510080 China; 2 Department of Vascular Surgery, the Second Xiangya Hospital of Central South University, 139 Renming Middle Road, Changsha, 410011 China; 3 Department of Vascular Surgery, National-Guangdong Joint Engineering Laboratory for Diagnosis and Treatment of Vascular Diseases, the First Affiliated Hospital of Sun Yat-sen University, 58 Zhongshan Road 2, Guangzhou, 510080 China

**Keywords:** Catheter-directed thrombolysis, Deep venous thrombosis, Low molecular-weight heparin, Dose of anticoagulation therapy

## Abstract

**Background:**

The optimal anticoagulant scheme during catheter-directed thrombolysis (CDT) for deep venous thrombosis (DVT) remains unknown. This study was performed to evaluate the feasibility of anticoagulation therapy using low molecular-weight heparin (LMWH) during CDT for DVT.

**Methods:**

The clinical data of DVT patients who underwent CDT during the past six years was retrospectively collected and reviewed. Patients were divided into therapeutic-dose anticoagulation (TPDA) and sub therapeutic-dose anticoagulation (sub-TPDA) groups according to LMWH dosage.

**Results:**

A total of 61 patients involving 61 limbs were comprised. Acute and subacute DVT were identified in 39 (63.9%) and 22 (36.1%) patients, respectively. Thrombosis involving the iliac vein was identified in 34 (55.7%) patients. Inferior vena cava filter placement was performed in 38 (62.3%) patients. Intraoperatively, adjunctive balloons, stents, and thrombectomy were provided for nine (14.8%), four (6.6%), and one (1.6%) patients, respectively. Twenty (32.8%) patients accepted TPDA therapy, while 41 (67.2%) patients were administrated with sub-TPDA therapy. Median urokinase infusion rate was 2.5 (0.83 to 5) × 10^4^ U/h. Median infusion duration time was 4 (2 to 14) days, and median urokinase dose infused was 2.4 (0.6 to 10.80) × 10^6^ U. During CDT, five (8.2%) cases of minor bleeding were observed, and blood transfusion was not required. No major bleeding, symptomatic pulmonary embolisms, or death occurred. Complete (> 90%) and partial thrombolysis (50 ~ 90%) were achieved in 56 (91.8%) patients. In comparison with sub-TPDA group, TPDA group exhibited no significant differences in baseline characteristics, clinical improvement, thrombolysis results, and complications.

**Conclusions:**

Anticoagulation therapy using low molecular-weight heparin during CDT with low infusion rate for DVT is likely to be feasible and safe. Sub-therapeutic-dose anticoagulation and therapeutic-dose could be used for CDT with similar clinical outcome and bleeding complications.

## Introduction

Deep venous thrombosis (DVT) is a common disease with an incidence of approximately 1 ~ 2 per 1000 persons per year [1]. It is one of the major causes of pulmonary embolism (PE), which led to nearly 1 60,000 deaths in American during the period from 1998 to 2018 [2].

Although anticoagulation therapy has proven to be effective and safe in preventing PE and recurrence of DVT and improving patients’ quality of life [3, 4], DVT management is still facing challenging since anticoagulation alone does not resolve the thrombus formed in the vein. Consequently, approximately 25 ~ 50% of proximal DVT patients develop post-thrombotic syndrome (PTS) because of valve incompetence and long-term venous hypertension [5].

Catheter-directed thrombolysis (CDT) has been proposed for symptomatic patients with severe DVT, particularly in the setting of phlegmasia alba dolens [6]. Numerous studies reported the clinical benefit of CDT in the treatment of symptomatic DVT [7]. Despite the increased interest in CDT, consensus opinion has not been reached regarding the optimal anticoagulant scheme during CDT, including the use of low molecular-weight heparin (LMWH), safety and effectiveness of therapeutic-dose anticoagulation (TPDA) versus sub-therapeutic-dose anticoagulation (sub-TPDA) during CDT [8, 9].

In the past six years, anticoagulation therapy with LMWH was used during CDT at our institution. This study was performed to evaluate the feasibility of LMWH for CDT and explore the optimal anticoagulation dose of LMWH during CDT.

## Methods

### Data collection

This retrospective study was approved by the Institutional Review Board and was performed in the Department of Cardiovascular Surgery at the Sun Yat-sen Memorial Hospital of Sun Yat-sen University. Patients who underwent CDT at our institution during the period from January 2014 to December 2019 were included. Informed consent was obtained from involved patients. Patients were diagnosed with DVT according to clinical features and ultrasounds. Clinical data including demographics, co-morbidities, risk factors, ultrasound reports, venography reports, operative notes, and complications were tabulated.

### Diagnosis

DVT was diagnosed according to clinical manifestations, the level of D-dimer, and ultrasound results. Only patients with iliac or femoral vein thrombi were included. DVT patients with duration time (calculated from onset of symptoms) ranging from 14 days to one month were classified as subacute DVT.

### Definition of variables

Efficacy outcomes included thrombolysis degree, clinical improvement, and mid-thigh and mid-crus circumferences after CDT. Thrombolytic efficacy of CDT was confirmed by color doppler ultrasound or venography. A scoring system mentioned by Mewissen et al. [7] was used for evaluating thrombolytic outcome in this study. Complete thrombolysis was defined as > 90% thrombus removal, and few clots were found after the procedure. Partial thrombosis was defined as 50–90% thrombosis removal. Clinical improvement was defined as a significant decrease in pain and/or swelling of the affected extremity during hospitalization. Mid-thigh circumferences were measured 15 cm above the upper margin of the patella, while mid-crus circumferences were 10 cm below the lower margin of the tibial tuberosity.

Safety outcomes comprised CDT-related complications during hospital stays, including major and minor bleeding, symptomatic PE, and death. Major and minor bleeding were defined as described [10]. Systematic PE and intracranial hemorrhage were diagnosed with computer tomography, which was given for patients with signs of PE (anhelation, hyoxemia, etc.) or intracranial hemorrhage (unconsciousness, powerlessness).

### Groups

CDT was performed by two surgical teams, and they provided anticoagulation treatment with different regimens of LMWH. Patients were divided into TPDA and sub-TPDA groups according to LMWH dosage.

### Anticoagulation therapy

All patients accepted a weight-based (1 mg/kg) twice-a-day regimen of LMWH (Lovenox; Sanofi, Paris, France) before and after CDT. During CDT, for the sub-TPDA group, LMWH were given at a fixed-dose of 40 mg every 12 h, while the TPDA group was administered the same weight-based (1 mg/kg) twice-a-day regimen.

### Catheter-directed thrombolysis

A recyclable inferior vena cava filter (OptEase (Cordis, USA) or Celect (Cook, USA)) was generally recommended for patients with high risk of PE [1]:. previous PE [2]; planning to accept pneumatic compression treatment. The filter was implanted via the healthy femoral or jugular vein before CDT, and it was removed when the CDT ended.

Retrograde catheterization of the femoral vein in the healthy lower extremity or antegrade catheterization of the popliteal vein in the affected lower extremity was performed. A 4F or 5F multi-sidehole infusion catheter (UniFuse, (Angiodynamics, USA)) was advanced. The tip of the infusion catheter was placed within the thrombus, and its position changed according to ultrasound or venography. The length of the lateral-hole segment for placement into the thrombus was selected based on thrombus distribution. Dose of urokinase (Livzon Pharmaceutical Group, Inc., China) was calculated according to weights of patients. Urokinase was first injected at a bolus dose of 2 ~ 3 × 10^5^U. Then urokinase was continuously infused with a dose of 1 ~ 1.5 × 10^4^ U/kg/d. In other words, the urokinase was given with a dose of 4 ~ 12 × 10^5^ U per day (the weight ranged from 40 to 80 kg) [11]. In rare cases with high risks of bleeding, the estimated dose might be reduced by half [12]. The risks of bleeding were evaluated based on the guideline developed by American College of Chest Physicians [13].

Coagulation function was tested daily. Infusion rate of urokinase dosage was adjusted according to Fibrinogen (FIB) concentration: infusion rate slowed by 50% if plasma FIB concentration decreased to < 1.5 g/L; CDT was suspended and restarted with a rate slowed down by 50% if plasma FIB concentration dropped to < 1.0 g/L.

Residual thrombus was evaluated daily with ultrasound or venography. For patients who experienced complete thrombolysis, CDT was discontinued. For patients who experienced partial thrombolysis, CDT was discontinued if patients met at least one criterion [1]: serum level of D-dimer exhibited no significant change [2]; complications with bleeding [3]; ultrasound or venography indicated no improvement. During, the catheter position might be adjusted according to thrombolytic outcome. Generally, thrombolytic duration time should be less than seven days.

Adjunctive balloons and stents were used for cases with iliac vein compression or residua stenosis after CDT. During CDT, patients were put on bedrest. The affected limb was elevated and extracts of horse chestnut seeds’ tablets (Aescuven forte, CesraArzneimittelGmbll&CoKG, Germany) were used to alleviate swelling. Pneumatic compression treatment was conserved for patients who accepted ICF implantation during CDT. Elastic compression stockings were given for patients after CDT.

### Management of bleeding

If patients manifested a major bleeding event, CDT was discontinued. FIB, prothrombin complex, or fresh frozen plasma was given. Proton-pump inhibitors were administrated for patients who experienced a gastrointestinal bleeding event. If patients had a minor bleeding event, CDT was suspended and resumed at a reduced dosage if the minor bleeding could be controlled. If the minor bleeding continued, CDT was discontinued permanently.

### Statistical analysis

The continuous variables were expressed as the mean (standard deviation) or median (range), whereas the categorical variables were recorded as the number and percentage. A *P* value < 0.05 indicated a significant difference. Continuous data was analyzed with analysis of variance, paired t tests or Mann-Whitney testing, and categoric variables with Chi-square test or Fisher’s exact probabilities.

## Results

A total of 61 patients containing 61 limbs were treated. The average age was 56.2 years old, ranging from 21 to 88. Thirty male patients and 31 females were involved. Nine patients were addicted to smoking. Thirteen patients had a history of hypertension, and five patients were diagnosed with diabetes. Clinical characteristics are shown in Table 1.

**Table 1:** Clinical characteristics of 61 cases of DVT patients experiencing catheter-directed thrombosis

Variables	No.(%) or median (range)
Patents	61
Age, year	56.2 (21 to 88)
Female	31 (50.8)
Weight, kg	61.5 (43 to 82)
Smoking	9 (14.8)
Symptom duration	
Acute (0-2w)	39 (63.9)
Subacute (2w-1 m)	22 (36.1)
Risk factors	
Surgery within last 30 days	10 (16.4)
Immobilization	7 (11.5)
Malignancy	8 (13.1)
Childbirth	1 (1.6)
Trauma	5 (8.2)
Oral contraceptive use	1 (1.6)
Previous DVT or PE	3 (4.9)
Hypercoagulable state	2 (3.3)
Cockett sydrome	2 (3.3)
Unknown	22 (36.1)
Involving iliac vein	34 (55.7)
Co-existing PE	4 (6.6)
Pre-operative mid-thigh circumference, cm	45.8 (4.9)^a^
Pre-operative mid-crus circumference, cm	36.9 (3.4)^b^

*Note: w = week, m = month, DVT = deep venous thrombosis, PE = pulmonary embolism,* ^*a*^*10 cm from lower margin of the tibial tuberosity;* ^*b*^*15 cm from upper margin of the patella*

Twenty-two (36.1%) patients were classified as subacute DVT. Surgery within the past 30 days was the leading cause of DVT. Risk factors of 22 patients were unknown. Thrombosis involved iliac vein was identified in 34 (55.7%) patients. The remaining 27 patients had femoropopliteal venous thrombosis. Co-existing PE was found in four (6.6%) patients.

Preoperative inferior vena cava filter placement was placed in 38 (62.3%) patients. Intraoperatively, adjunctive balloons (Mustang, Boston Scientific, American), stents (Wallstent, Boston Scientific, American), and percutanous mechanical thrombectomy (AnjioJet, Boston Scientific, American) were provided for nine (14.8%), four (6.6%), and one (1.6%) patients, respectively. Twenty (32.8%) patients accepted TPDA therapy, while 41(67.2%) patients were administrated sub-TPDA therapy. Median urokinase infusion rate was 2.5 (0.83 to 5) × 10^4^ U/h. Median infusion duration time was 4 (ranged 2 to 14) days, and median dose infusion was 2.4 (0.6 to 10.80) × 10^6^ U. Among these, 3 patients accepted thrombolytic treatment with the dose of urakinase reduced by half because of recent surgery or active bleeding: two cases of minimally invasive surgery and one renal hemorrhage that had been managed with selective arterial embolization of renal artery.

During thrombolytic therapy, five (8.2%) cases of minor bleeding were identified, and no blood transfusion was required. No major bleeding, symptomatic PE, intracranial hemorrhage, or deaths occurred. Five patients experienced a minor bleeding. Among these, four female patients and one male patient were involved, with a median age of 64 (46 to 80) years. Median thrombotic duration time was seven (four to 12) days. Median infusion rates were 3.75 (2.5 to 4.2) × 10^4^ U/h, and median urokinase dosage was 4. (1.8 to 9.9) × 10^6^ U. Compared with patients without bleeding, patients who experienced bleeding were given more urokinase (*P* = .029), and the urokinase was infused at faster rate (*P* = .007). The cases with bleeding were managed by slowing down the infusion rate and suspending CDT. Intervention strategies are described in Table 2.

**Table 2:** Intervention strategies of 61 cases of catheter-directed thrombosis

Variables	No.(%) or median (range)
Balloon	9 (14.8)
Stent	4 (6.6)
Percutanous mechanical thrombectomy	1 (1.6)
Inferior vena cava filter placement	38 (62.3)
Aspiration using catheter	2 (3.3)
Low molecular heparin	
Therapeutic dose	23 (37.7)
Subtherapeutic dose	38 (62.3)
Median urokinase dose, 10,000 U	240 (60 to 1080)
Median infusion rates, 10,000 U/hour	2.5 (0.8 to 5)
Median duration time, day	4 (2 to 14)

As for coagulation function, a plasma FIB concentration < 2.0 g/L was found in eight patients. FIB concentration < 1.5 g/L was identified in two of these patients. The infusion rate of urokinase was slowed down in these two patients. No FIB was infused.

After CDT, complete and partial thrombolysis was achieved in 56 (91.8%) patients, including all patients with acute DVT and 17 (77.3%) patients with subacute DVT. Patients with less than 50% thrombosis removal were all classified as subacute DVT. Among these, median urokinase dosage was 1.8 (1.6 to 6) × 10^6^ U. The mid-thigh (45.8 ± 4.9 vs. 43.6 ± 4.5, *P* < .01) and mid-crus (36.9 ± 3.4 vs. 33.9 ± 2.5, P < .01) circumference (cm) significantly decreased after CDT. Clinical improvement was confirmed in 57 (93.4%) patients. Clinical outcome is shown in Table 3.

**Table 3:** Clinical outcome of 61 cases of DVT patients underwent catheter-directed thrombolysis

Variables	No.(%) or mean (standard deviation)
Thrombolysis degree	
Complete	13 (21.3)
Partial	43 (70.5)
Clinical improvement	57 (93.4)
Post-eroperative mid-thigh circumference	43.6 (4.5)^a^
Post-eroperative mid-crus circumference	33.9 (2.5)^b^
Complications	
Bleeding	5 (8.2)
Minor bleeding	5 (8.2)
Errhysis at the puncture site	1 (1.6)
Dermal ecchymosis	2 (3.3)
Menorrhagia	2 (3.3)
Major bleeding	0 (0)
PRBC transfusion	0 (0)
Death	0 (0)
Allergy	1 (1.6)

*Note:* ^*a*^*10 cm from lower margin of the tibial tuberosity;* ^*b*^*15 cm from upper margin of the patella. PRBC = packed red blood cells*

In comparison with sub-TPDA group, TPDA group did not exhibit significant difference in demographic characteristics, lesion characteristics, use of urokinase, and adjunctive strategies. Mid-thigh and mid-crus circumference, clinical improvement, rate of complete and partial thrombolysis, and bleeding were similar between the two groups. Comparison of outcomes are described in detail in Table 4.

**Table 4:** Comparison outcome of therapeutic dose group and sub-therapeutic dose group

Variable	TPDA group	sub-TPDA group	P value
Number	23	38	–
Female	9 (39.1)	22 (57.9)	0.155
Age	57.4 (17.3)	54.3 (13.7)	0.466
Acute DVT	15 (65.2)	24 (63.2)	0.871
Subacute DVT	8 (34.8)	14 (36.8)	
Thrombolytic treatment			
Median infusion rates 10,000 U/hour	2.5 (0.8–4.2)	2.5 (1.7–5)	0.891
Median dose, 10,000 U	240 (60–1080)	240 (80–480)	0.456
Median duration time, day	4 (2–12)	4 (2–14)	0.131
Balloon	3 (13.0)	3 (7.9)	1.000
Stent	1 (4.3)	6 (15.8)	0.236
Thrombosis degree			
Undissolved	3 (13.0)	2 (5.3)	0.665
Partial	15 (65.2)	28 (73.7)	
Complete	5 (21.7)	8 (21.1)	
Clinical improvement	20 (87.0)	37 (97.4)	0.146
Decreased mid-thigh circumference, cm	2.1 (2.4)	2.4 (1.8)	0.716^a^
Decreased mid-crus circumference, cm	2.2 (1.9)	3.1 (2.2)	0.268^b^
Bleeding			
Major bleeding	0 (0)	0 (0)	1.000
Minor bleeding	1 (4.3)	4 (10.5)	0.641

*Note: w = week, m = month, DVT = deep venous thrombosis,* ^*a*^*10 cm from lower margin of the tibial tuberosity;* ^*b*^*15 cm from upper margin of the patella*

## Discussion

Although various strategies were applied for removing thrombus, CDT remained the mainstream therapeutic strategy. CDT therapy could not only reduce mechanical trauma to the vessel wall compared with an open balloon thrombectomy procedure, but also manage thrombus in smaller distal vessels that are generally not accessible by a thrombectomy catheter [14]. In clinical practice, CDT therapy was increasingly used in combination with percutaneous mechanical thrombectomy, creating a pharmacomechanical thrombectomy system.

Unfractionated heparin was preferentially used for anticoagulation therapy during CDT due to its shorter half-life and complete reversibility by using protamine. Though favorable results have been obtained, numerous studies have demonstrated that risks of bleeding related to CDT therapy were alarmingly high, particularly in elderly patients [7, 15, 16]. In comparison with unfractionated heparin, LMWH seemed to be equally effective and safer for venous thromboembolism [17]. In addition, unfractionated heparin was given by intravenous continuous infusion during CDT, while LMWH was easier to use by subcutaneous injection. However, the evidence of using of LMWH during CDT was limited.

In Chen et al. [12] study involving 46 patients with acute iliofemoral venous thrombosis, LMWH in combination with low dose urokinase was applied for CDT. Patients were divided into high-risk and low-risk groups according to their risk of bleeding. The high-risk group received CDT with a median infusion rate of 1.0 × 10^4^ U/h, while the low-risk group had a median infusion rate of 2.0 × 10^4^ U/h. The rate of complete thrombolysis and clinical improvement was consistent with studies using unfractionated heparin for CDT [18], and the rate of bleeding was lower.

A retrospective study performed by Graif et al. [19] included 45 patients accepting anticoagulation with LMWH during CDT for PE and 111 patients with unfractionated heparin. Graif et al. [19] found that therapeutic anticoagulation using LMWH during CDT for PE was safe. Their study did not find a significant difference between LMWH and unfractionated heparin with respect to hemorrhagic and general complication rates.

Favorable results were observed in the present study as well. Clinical improvement was achieved in all patients with acute DVT. The rate is acceptable in comparison with other studies [16]. For patients with subacute DVT, who were thought to be poorly responsive to CDT [20], complete or partial thrombolysis was also identified in nearly 75% of cases. In addition, a relatively low rate of adjunctive strategies, including balloons, stents, and percutanous mechanical thrombectomy, was applied, which might underestimate the thrombolytic result [21, 22].

In a meta-analysis involving 45 studies, major bleeding occurred in 196 (7.9%) of 2467 patients experiencing CDT, and 18 (0.8%) of 2388 patients underwent CDT developed intracranial bleeding [23]. No major bleeding or intracranial bleeding was identified in the present study. Minor bleeding occurred in 8.2% of patients. The rate of minor bleeding was lower compared with studies in which unfractionated heparin was used for anticoagulation therapy [22], which could be explained by relatively low infusion rate of urokinase. On coagulation function assay, FIB concentration < 1.5 g/L was identified in two patients, and it was reversible by suspending CDT. These results indicated that it is safer to use LMWH for CDT.

We respectively reviewed the infusion rate, urokinase dosage, and thrombolytic duration of patients who underwent minor bleeding. Patients with bleeding were found to have faster infusion rate and more urokinase. The results showed that the risks of bleeding increased as dosage and infusion rate increased. Based on our results, it is reasonable that thrombolytic duration time should not exceed seven days and total dose of urokinase should be less than 4 × 10^6^ U. Furthermore, given the infusion rate was relatively low in the present cohort, the conclusions should be carefully quoted and might be confined to CDT with low infusion rate.

The optimal dose of anticoagulation remained unclear during CDT. In comparison with sub-TPDA group, CDT with TPDA exhibited no significant difference in thrombolytic outcome or bleeding complications in the present study. The similar results could be explained as follows. Dose of urokinase and infusion rate had a higher impact on the clinical outcome, and the effect of anticoagulation therapy might be overshadowed. The relatively limited number of patients involved might affect the power of statistical tests. Based on these results, both sub-therapeutic and therapeutic dose LMWH could be used for anticoagulation therapy during CDT.

Serial hematocrit levels and coagulation function assays should be mandatory [24, 25], and they were performed daily in the present study. The frequency of testing was less than that accepting anticoagulation therapy with unfractionated heparin [26]. We observed that parameters of blood coagulation were stable during CDT. Anti-factor Xa level could be used for monitoring of LWMH. However, the optimal anti-factor Xa level was unknown during CDT [24, 26]. The assay was not available at that time in our center. Further studies evaluating optimal anti-factor Xa level during CDT should be performed.

### Limitations

First, the present study was based on retrospectively collected data and shared the same flaws as other observational studies. Second, follow-up outcome variables were absent in the present study. Outcome variables should be broadened to include follow-up outcome variables that were related with CDT, such as PTS and quality of life. Furthermore, regimen of CDT varied among patients, which might influence the reliability of conclusions.

## Conclusions

Anticoagulation therapy using low molecular-weight heparin during CDT with low infusion rate for DVT is likely to be feasible and safe. Sub-therapeutic-dose anticoagulation and therapeutic-dose could be used for CDT with similar clinical outcome and bleeding complications.

## Data Availability

The datasets used during the current study are available from the corresponding author on reasonable request.
